# Effects of Melatonin on Liver Injuries and Diseases

**DOI:** 10.3390/ijms18040673

**Published:** 2017-03-23

**Authors:** Jiao-Jiao Zhang, Xiao Meng, Ya Li, Yue Zhou, Dong-Ping Xu, Sha Li, Hua-Bin Li

**Affiliations:** 1 Guangdong Provincial Key Laboratory of Food, Nutrition and Health, School of Public Health, Sun Yat-sen University, Guangzhou 510080, China; zhangjj46@mail2.sysu.edu.cn (J.-J.Z.); mengx7@mail2.sysu.edu.cn (X.M.); liya28@mail2.sysu.edu.cn (Y.L.); zhouyue3@mail2.sysu.edu.cn (Y.Z.); xudp@mail2.sysu.edu.cn (D.-P.X.); 2 School of Chinese Medicine, The University of Hong Kong, Hong Kong 999077, China; u3003781@connect.hku.hk; 3 South China Sea Bioresource Exploitation and Utilization Collaborative Innovation Center, Sun Yat-sen University, Guangzhou 510006, China

**Keywords:** melatonin, effect, liver injuries, steatosis, fatty liver, hepatitis, fibrosis, cirrhosis, hepatocarcinoma

## Abstract

Liver injuries and diseases are serious health problems worldwide. Various factors, such as chemical pollutants, drugs, and alcohol, could induce liver injuries. Liver diseases involve a wide range of liver pathologies, including hepatic steatosis, fatty liver, hepatitis, fibrosis, cirrhosis, and hepatocarcinoma. Despite all the studies performed up to now, therapy choices for liver injuries and diseases are very few. Therefore, the search for a new treatment that could safely and effectively block or reverse liver injuries and diseases remains a priority. Melatonin is a well-known natural antioxidant, and has many bioactivities. There are numerous studies investigating the effects of melatonin on liver injuries and diseases, and melatonin could regulate various molecular pathways, such as inflammation, proliferation, apoptosis, metastasis, and autophagy in different pathophysiological situations. Melatonin could be used for preventing and treating liver injuries and diseases. Herein, we conduct a review summarizing the potential roles of melatonin in liver injuries and diseases, paying special attention to the mechanisms of action.

## 1. Introduction

The liver is a vital organ of the human body that is responsible for numerous fundamental and important roles, including digestive and excretory functions, in addition to nutrient storage and metabolic functions, synthesis of new molecules, and purification of toxic chemicals [1]. Recently, liver injuries induced by various factors, such as chemical pollutants, drugs, and alcohol, have been studied widely. Liver steatosis, fatty liver, hepatitis, fibrosis, cirrhosis and hepatocellular carcinoma are the most prevalent liver diseases, and have also been investigated extensively. The therapy choices for these injuries and diseases are very few. Therefore, it is imperative to seek an effective and safe treatment for liver injuries and diseases.

Melatonin (*N*-acetyl-5-methoxytryptamine) is mainly synthesized from the amino acid tryptophan by the pineal gland in mammals and humans [2,3]. Firstly, tryptophan is hydroxylated by tryptophan-5-hydroxylase to form 5-hydroxytryptophan. Then, it is decarboxylated to 5-hydroxytryptamine (serotonin) by l-aromatic amino acid decarboxylase. After serotonin acetylation, *N*-acetylserotonin is produced. At last, *N*-acetylserotonin is converted to *N*-acetyl-5-methoxytryptamine (melatonin) in the pineal gland [4]. Except for endogenous melatonin, exogenous melatonin can be consumed from a daily diet. There are lots of melatonin-rich foods, such as sour cherries, walnuts, and orange juice [5]. Melatonin could regulate the circadian rhythm, and alleviate insomnia and jet lag [5,6]. In addition, melatonin showed a variety of regulatory effects on sexual behavior, immune function, energy metabolism, the cardiovascular system, the reproductive system, and the neuropsychiatric system [4,7]. Melatonin also exhibited anticancer and anti-osteoarthritic activities. Moreover, melatonin showed strong antioxidant activity and possessed protective properties against oxidative stress [8,9]. Melatonin is the focus of many research areas due to its ability to scavenge free oxygen radicals and thereby protect cells and tissues from radical damage [10]. Recently, studies have focused on the roles of melatonin in oxidative stress, lipid metabolism, and its potential therapeutic action. There are numerous studies exhibiting the beneficial abilities of melatonin on liver injuries and diseases. This review summarizes the effects of melatonin on liver injuries induced by various factors and liver diseases, including liver steatosis, non-alcohol fatty liver, hepatitis, liver fibrosis, liver cirrhosis, and hepatocarcinoma, focusing on the mechanisms of action, such as antioxidant, anti-inflammation, anticancer, antiproliferation, and pro-apoptosis.

## 2. Protective Effects of Melatonin on Liver Injuries

### 2.1. Protective Effects of Melatonin on Chemical Pollutant-Induced Liver Injuries

Humans are exposed to highly variable chemical pollutants, which could result in harmful effects on the liver. The effects of melatonin on liver damage induced by chemical pollutants such as organic compounds, metals, and mycotoxins have been studied widely.

The experimental model of carbon tetrachloride (CCl_4_)-induced liver injury was frequently used in research on melatonin. CCl_4_ could induce acute or chronic liver damage. In acute liver injury induced by CCl_4_, liver lipid peroxide (LPO) content, malondialdehyde (MDA), lipid hydroperoxides (LOOH), and liver triglyceride (TG) contents were increased, and liver reduced glutathione (GSH) content, serum TG concentration, liver tryptophan 2,3-dioxygenase (TDO) activity, and serum albumin concentration were decreased [11,12]. In addition, it showed reductions in concentration of ascorbic acid (ASC), activities of superoxide dismutase (SOD), catalase (CAT), and glutathione reductase (GSSG-R), and increases in activities of G-6-PDH, xanthine oxidase (XO), and vitamin E concentration [13]. Apart from the changes in biochemical parameters, significant lipid and hydropic dystrophy of the liver, necrosis, fibrosis, mononuclear cell infiltration, hemorrhage, fatty degeneration, and formation of regenerative nodules were also observed in rats injected with CCl_4_ [14,15]. In addition, insulin-like growth factor I (IGF-I) expression observed in hepatocytes was weak in the CCl_4_ injection group [16]. Substantial impairment of mitochondrial respiratory parameters was caused by acute intoxication of CCl_4_ in the liver [17]. However, melatonin ameliorated the liver injury induced by CCl_4_. Reductions in concentration of hepatic ASC and activities of SOD, CAT, and GSSG-R and the increase in LPO content and hepatic XO activity were attenuated after melatonin administration (10, 50, or 100 mg/kg body weight (BW)) in a dose-dependent manner [11,13]. CCl_4_ could cause mitochondrial alterations via an oxidation of intramitochondrial GSH by 25% (*p* < 0.05), an inhibition of succinate dehydrogenase (complex II) by 35% (*p* < 0.05) and a rise of blood plasma nitric oxide (NO) level by 45% (*p* < 0.05). Melatonin (10 mg/kg BW) reversed the increase in mitochondrial GSH peroxidase (GSH-Px) activity and prevent the elevation of NO level in plasma but not protect mitochondrial functions [18]. Furthermore, CCl_4_-induced upregulation of tumor necrosis factor-alpha (TNF-α) and programmed cell death-receptor (Fas) mRNA expression was significantly restored by melatonin treatment at the concentration of 10 mg/kg BW [19]. Melatonin also increased IGF-I expression at a dose of 25 mg/kg BW, and membrane rigidity and protein oxidation were fully prevented by melatonin at 10 mg/kg BW [16]. Morphological and histopathological changes induced by CCl_4_ were restored after melatonin (10 or 25 mg/kg BW) treatment in rats [14,20]. The chronic liver injury induced by CCl_4_ was less studied than acute injury. Liver MDA content was considerably increased, and SOD and GSH-Px activities were meaningfully decreased in rats administrated with CCl_4_ chronically. Moreover, it triggered an obvious elevation in apoptotic cells. After administration of melatonin (25 mg/kg BW), an increased level of MDA and decreased activities of SOD and GSH-Px were restored, and CCl_4_-induced apoptosis was markedly reduced [21].

Benzene and toluene are common organic chemical pollutants. Both have detrimental effects on humans and animals. Benzene could cause liver function impairments and the lipid peroxidation of mitochondria and microsome [22,23]. The protective effects of melatonin on liver injury induced by benzene were identified. Hepatosomatic indices, bilirubin as well as hydroxyproline in male and female rats treated with benzene were significantly lowered after 30 days’ melatonin treatment (0.25 mL of 2% melatonin) [22]. Mitochondrial and microsomal lipid peroxidation was inhibited by melatonin at the concentration of 10 mg/kg BW. The activity of cytochrome P_450_2E1 (CYP_450_2E1), which is responsible for benzene metabolism, declined after 15 days’ melatonin treatment, but it rose again, though not significantly, after 30 days’ treatment with melatonin in the benzene-treated groups. The results showed that melatonin affected CYP_450_2E1 and protected against lipid peroxidation induced by benzene [23]. The harmful effects of toluene on animals were investigated too. Serum ALT, aspartate transaminase (AST), and tissue MDA were considerably increased, and serum albumin was decreased in toluene-inhaled rats. Massive hepatocyte degeneration, ballooning degeneration, and mild pericentral fibrosis were detected in toluene-inhaling rats. The reactivity of Bax immune increased markedly. After melatonin treatment (10 mg/kg BW), the increase in tissue MDA, serum ALT and AST levels was significantly reduced, and balloon degeneration, fibrosis, and Bax immune reactivity were inhibited in the livers of toluene-inhaling rats [24].

Cadmium (Cd) is one of the most toxic substances found in the environment. It is well known that Cd could induce hepatotoxicity in humans and multiple animal models [25]. The animals received subcutaneous injections of cadmium chloride at 1 mg/kg BW dose showed significantly higher MDA levels and reduced activity of SOD (*p* < 0.05). Treatment with 10 mg/kg BW melatonin caused a substantial decrease in MDA when compared to non-treated animals (*p* < 0.05) and an increase in the SOD activity that was almost the same as the controls [26]. Moreover, exposure to Cd induced diverse histopathological changes, including loss of normal structure of the parenchymatous tissue, cytoplasmic vacuolization, cellular degeneration and necrosis, congested blood vessels, destructed cristae mitochondria, fat globules, severe glycogen depletion, and lipofuscin pigments, which could be counteracted by melatonin treatment [27]. Cd exposure produced cytotoxicity, disturbed the mitochondrial membrane potential, increased reactive oxygen species (ROS) production, and reduced mitochondrial mass and mitochondrial DNA content. Consistently, Cd exposure decreased expression and activity of sirtuin 1 protein and stimulated acetylation of PGC-1α, which is a vital enzyme associated with mitochondrial biogenesis and function [28]. Accumulation of Cd in the liver induced oxidative stress and inflammation. Melatonin reduced liver injury and inflammation through decreasing serum ALT/AST levels, inhibiting pro-inflammatory cytokine production, preventing NOD-like receptor pyrin domain containing 3 (NLRP3) inflammasome activation, ameliorating oxidative stress, and attenuating hepatocyte death. In vivo and in vitro, Cd-induced TXNIP overexpression was markedly abrogated and the interaction between TXNIP and NLRP3 was decreased by melatonin [29]. In addition, melatonin increased hepatic GSH levels and improved histopathological changes after Cd^2+^ exposure. In addition, melatonin prevented lipid peroxidation induced by Cd^2+^. Also, melatonin reduced metal-induced oxidative injury because of its chelating property [30]. Melatonin treatment efficiently attenuated Cd-induced mitochondrial oxidative injuries. Moreover, melatonin stimulated PGC-1α and improved mitochondrial biogenesis and function [28]. Additionally, Cd induced mitochondrial-derived superoxide anion-dependent autophagic cell death. Explicitly, the expression and activity of sirtuin 3 protein were decreased and the acetylation of SOD2, a critical enzyme associated with mitochondrial ROS production, was promoted, leading to reduced activity [25]. Melatonin treatment showed protective effects by enhancing the activity of sirtuin 3, decreasing the acetylation of SOD2, inhibiting production of mitochondrial-derived O_2_^•−^ and suppressing the autophagy induced by 10 μM Cd. In addition, Cd-caused autophagic cell death could be prevented by melatonin via increasing sirtuin 3 activity in vivo [25]. Lead also induced hepatic toxicity. The increased LPO and decreased SOD, GSH, nuclear area (NA), nuclear volume (NV), and nuclear volume/cellular volume (N/C) were observed in the organs of rats treated with lead. Histopathological observations exhibited severe impairment in the liver and kidney of lead-treated rats. The increase of LPO was attenuated and the activity of SOD and level of GSH as well as the values of NA, NV, and N/C were restored by melatonin administration. Furthermore, the morphological damages in the liver and kidney were decreased and the tissues recovered [31].

Mycotoxins are secondary metabolites produced by certain toxigenic fungi; the common species are aflatoxins, fumonisins, trichothecenes, ochratoxin A, patulin, and zearalenone [32]. Among these mycotoxins, the aflatoxins and ochratoxin A were frequently used to induce liver injuries in research. It is well known that aflatoxins could produce chronic carcinogenic, mutagenic, teratogenic, and acute inflammatory effects [33]. The caspase-3 activities (apoptotic marker) and heat shock protem-70 (HSP70) were significantly increased after aflatoxin B1 administration in rats. Moreover, the levels of MDA, oxidative stress indices, LPO, and NO in liver tissues were markedly increased, while GSH and Zn levels as well as GSH-Px and glutathione reductase enzyme activities in the liver were markedly reduced in aflatoxin-B1-treated rats [34,35]. Melatonin had beneficial effects on liver injury induced by aflatoxin B1. The apoptotic rate was significantly reduced after melatonin treatment. Caspase-3 activity, LPO, MDA and NO levels, and HSP70 expression were meaningfully reduced, while GSH and Zn levels and GSH-Px, GR, and glutathione-S-transferase (GST) activities were markedly improved because of melatonin administration [34,35]. Hepatic antioxidant and detoxification system were improved by melatonin treatment, therefore decreasing the apoptotic rate and the necrobiotic changes in the liver of rats [34]. Moreover, a significant increase (*p* < 0.05) in serum interleukin 1-β (IL-1β) was observed, which was correlated with hemorrhages and leucocytic and lymphocytic infiltration in the liver and intestines. Treatment with melatonin yielded a significant decrease (*p* < 0.05) in level of IL-1β. Melatonin showed considerable protection of hepatic tissues [33]. Ochratoxin A (OTA) is ubiquitous as a natural contaminant of moldy food and feed [36]. In rats treated with OTA, the LPO level in serum as well as LPO, MDA, and hydroxyproline levels in the liver and kidneys were higher than those of control rats. Concomitantly, the GSH level and SOD, CAT, GSH-Px, and GR activities in the liver and kidneys were markedly reduced [37,38]. Melatonin attenuated the change of LPO level in the serum, liver, and kidneys. In addition, the activities of GSH-Px, GR, and GST in the liver and kidneys were substantially improved in rats that were administrated melatonin. However, MDA and hydroxyproline levels in the liver and kidneys markedly decreased after the administration of melatonin [37,38]. Substantial histopathologic changes were also observed in the kidneys and livers of rats administrated OTA, which were reduced by the administration of melatonin [39]. Melatonin also had protective effects on OTA toxicity via inhibition of oxidative damage and fibrosis, and improved GST activity in both the liver and kidneys [37,38].

α-Naphthylisothiocyanate (ANIT) is a well-characterized biliary epithelial toxicant [40]. Cholestatic liver injuries of experimental rats were commonly induced by ANIT. In rats treated with ANIT only, liver injury with cholestasis appeared at 24 h after injection, judging from the serum levels of marker enzymes (ALT, AST, lactate dehydrogenase, γ-glutamyl transpeptidase, and alkaline phosphatase) and components (sera total bilirubin and total bile acids). In ANIT-treated rats, the formation of liver injury with cholestasis was dose-dependently inhibited by the administration of melatonin (10 or 100 mg/kg BW) at 12 h after ANIT treatment, mainly through preventing the progression of liver cell damage [41,42]. Moreover, in rats treated with ANIT alone, serum LPO concentration was improved at 24 h, while liver LPO concentration was improved at 12 h and further improved at 24 h. ANIT also caused myeloperoxidase (MPO) activity, an index of tissue neutrophil infiltration, elevating at 12 h after injection and further elevating at 24 h in the liver. The increases of LPO concentrations in the serum and liver and MPO activity in the liver were attenuated by oral administration of melatonin (10 or 100 mg/kg BW) in rats injected with ANIT [41]. Additionally, melatonin exhibited beneficial effects on ANIT-induced acute liver injury via decreasing the disorder of hepatic antioxidant defense systems. ANIT-treated rats showed several changes in hepatic antioxidant enzyme (Cu-SOD, Zn-SOD, CAT, Se-GSH-Px, and GSSG-R) activity, while melatonin (100 mg/kg BW) attenuated these changes [43]. The protective effect of melatonin, related indoles (6-hydroxymelatonin and *N*-acetylserotonin), and α-tocopherol against ANIT-induced liver injury was identified and compared in rats. It has shown that 6-hydroxymelatonin and *N*-acetylserotonin were less effective than melatonin in providing protection to liver injuries induced by ANIT. Melatonin administration reduced the severity of morphological alterations and prevented liver neutrophil infiltration, a key factor in the pathogenesis of ANIT-induced liver injury. 6-Hydroxymelatonin was unable to reduce neutrophil infiltration, while *N*-acetylserotonin only showed antioxidant effects but possessed no abilities to attenuate ANIT-induced hepatic damage in experimental conditions [44]. When compared with α-tocopherol, melatonin showed protective effects on both liver cell damage and biliary cell damage in ANIT-injected rats with cholestasis, while α-tocopherol showed protective effects on liver cell damage only. Moreover, the treatment of α-tocopherol increased α-tocopherol concentration in the liver and serum and weakened the elevated hepatic lipid peroxide level, MPO activity, and serum non-esterified fatty acid concentration. In comparison, melatonin treatment attenuated the increase of hepatic lipid peroxide level, MPO activity, serum α-tocopherol, non-esterified fatty acid, TG, and total cholesterol levels, with no effect on the hepatic α-tocopherol level [45]. Obviously, the beneficial effects of orally administered melatonin against ANIT-induced hepatotoxicity in rats were more powerful than those of α-tocopherol.

The effects of melatonin on liver injuries induced by other toxins not mentioned above are summarized in Table 1.

### 2.2. Protective Effects of Melatonin on Drug-Induced Liver Injuries

Drugs could induce liver injuries when taken at an overdose, or even at therapeutic doses in susceptible individuals [53]. Hepatotoxicity could be induced by several kinds of medicines, including anti-tumor, immunosuppressive, antiepileptic, anti-depressed, anxiolytic, antalgic drugs, and so on.

Adriamycin (ADR) is a drug used clinically for cancer treatment. However, it could cause adverse effects on the liver [54]. The GSH level in the liver cells was significantly reduced after administration of ADR in mice. Lipid peroxidation was also observed in mice treated with ADR [55]. Moreover, ADR caused excessive production of ROS and decreased activities of CAT, SOD, GSH-Px, GR, and MPO [56]. Melatonin had protective effects on hepatotoxicity induced by ADR in rats. The decrease in GSH concentration was significantly prevented and the activities of the enzymes mentioned above were improved by melatonin treatment [55,56]. Additionally, histopathological alterations reflecting hepatic dysfunction were significantly improved by melatonin [57]. Other anti-tumor drugs, such as methotrexate and letrozole, could also induce hepatotoxicity in rats. Increased MDA level and MPO activity and decreased GSH level were observed in the blood, liver, and kidneys of rats injected with methotrexate [58]. In addition, serum enzymes (ALT, AST, and ALP) were significantly increased, and necrotic hepatocytes with small crushed nuclei, portal space with severe inflammation, as well as hepatocytes surrounded by lymphocytic infiltration were observed in rats injected with cyclophosphamide [59]. In addition, letrozole, an aromatase inhibitor, was used to treat breast cancer. In female rats, hepatic function parameters such as AST, LDH, ALP, and bilirubin increased and mild histological changes in liver tissue were observed after the administration of letrozole [60]. All these changes induced by letrozole were improved or reversed by melatonin.

Immunosuppressive drugs can prevent graft rejection and autoimmune diseases. Cyclosporine A (CsA) is an extensively used immunosuppressive drug [61]. However, the treatment induces a lot of side effects, including nephrotoxicity, cardiotoxicity, hypertension, and hepatotoxicity. CsA-induced hepatotoxicity was characterized by histopathological changes, such as cytoplasmic vacuolization, dilatation of the sinusoids, apoptosis, many mitotic figures, alterations in GSH and MDA concentrations, and an increase in stress protein expression [61,62]. Additionally, tacrolimus is a powerful immunosuppressive agent that could modulate neutrophil infiltration during inflammation [63]. However, it had negative effects on the liver. The MDA, TNF-α, IL-6, and NO levels were increased in rats after injection with tacrolimus. Not surprisingly, these changes were reversed by melatonin treatment [63].

Psychiatric and neurological agents usually had side effects on patients. Carbamazepine is an antiepileptic drug that is adapted to a broad spectrum of psychiatric and neurological disorders [64]. Carbamazepine was identified to have side effects of hepatotoxicity. Oxidative stress is a potential mechanism for carbamazepine-induced hepatotoxicity [65]. In cells treated with 400 µM carbamazepine, oxidative stress, elevated ROS formation, LPO products, and a reduced mitochondrial membrane potential were observed. Cellular GSH content was decreased and oxidized GSH levels were elevated by carbamazepines. It has been demonstrated that melatonin showed powerful antioxidant effects on the hepatotoxicity caused by carbamazepine [65]. Phenytoin and phenobarbital are antiepileptic drugs too. Phenobarbital is the first-line choice for neonatal seizures treatment [66]. Both medicines induced hepatotoxicity. Phenytoin caused an increase in ROS formation, a reduction in intracellular reduced glutathione, an improvement of cellular oxidized glutathione, an enhancement of LPO, and mitochondrial impairment. The intensity of cellular injury was decreased by melatonin treatment [67]. In addition, the hepatotoxicity induced by phenobarbital was decreased by melatonin treatment through reducing (*p* < 0.01) the lipid peroxidation level and the rate of DNA synthesis, and increasing the cell cycle time [68]. Additionally, the liver damage induced by other three common pharmaceuticals used to treat psychiatric conditions has been investigated. Diazepam is a classical anxiolytic drug [69]. Oxidative stress was a possible molecular mechanism of the harmful effects associated with long-term diazepam administration. Melatonin as an antioxidant could attenuate the liver damage induced by diazepam. The increase of DNA synthesis and LPO were attenuated and the levels of GSH and SOD activity were restored by melatonin [70]. Trazodone is an FDA-approved antidepressant [71]. Trazodone was cytotoxic and caused cell death with LC_50_ of 300 µM within 2 h. In rat hepatocytes, ROS formation, MDA accumulation, GSH, and GSSG were increased, but mitochondrial membrane potential was decreased by trazodone administration. Administration of melatonin reduced the toxic effects of trazodone on isolated rat hepatocytes [72]. Moreover, chlorpromazine is an aliphatic phenothiazine, and is one of the typical antipsychotic drugs [73]. The possible beneficial effects of melatonin against chlorpromazine-induced liver injury in rats were identified. Melatonin meaningfully weakened the oxidative stress parameters, including lowering the MDA level in tissue homogenate while not changing the GSH level. In addition, serum activities of ALT, AST, and serum bilirubin were restored through pre-treatment and post-treatment with melatonin [74].

Acetaminophen (APAP) is a recognized analgesic and antipyretic drug. It is recognized to be safe when administered within its therapeutic range, but in cases of acute intoxication, hepatotoxicity can occur [75]. APAP hepatotoxicity is characterized by an extensive oxidative stress [76]. The effects of melatonin on APAP-induced liver injury have been studied. Pre-treatment with melatonin (50 or 100 mg/kg BW) inhibited the elevation in plasma ALT and AST activities in a dose- and time-dependent manner. In addition, centrilobular hepatic necrosis with inflammatory cell infiltration and elevations in hepatic LPO and MPO activity and release of NO and IL-6 into blood circulation were remarkably inhibited by melatonin treatment (100 mg/kg BW) at 4 h before APAP administration [77]. Moreover, APAP-induced activation of the serine/threonine kinase receptor interacting protein 1 (RIP1) was significantly attenuated by melatonin. In addition, APAP-induced hepatic c-Jun *N*-terminal kinase (JNK) phosphorylation, mitochondrial Bax translocation and translocation of apoptosis-inducing factor (AIF) from mitochondria to nuclei were all prevented by melatonin. It could be concluded that melatonin protected against AIF-dependent cell death via its direct prevention of hepatic RIP1 and following JNK phosphorylation and mitochondrial Bax translocation during the acute liver failure induced by APAP [78]. Interestingly, although APAP-induced liver injury was primarily caused by CYP_450_2E1-driven conversion of APAP into hepatotoxic metabolites, no alterations were produced by melatonin on hepatic CYP2E1 expression [78].

### 2.3. Protective Effects of Melatonin on Alcohol-Induced Liver Injury

Consumption of alcohol is rapidly increasing in the world. Alcoholic consumption is consistently linked with the development of several health problems, such as cancer, cardiovascular diseases, diabetes mellitus, obesity, liver damage, alcoholic hepatitis, liver cirrhosis, and hepatocarcinoma [79,80,81], for which the liver is the most adversely affected organ [82]. Chronic treatment with alcohol increased AST, ALT and total bilirubin, TG, and MDA levels, and decreased total liver protein [83]. Melatonin possesses various biological and physiological actions. There are several studies exploring the effects of melatonin on alcohol-induced hepatic injury. The serum aminotransferase level, hepatic cell damage, steatosis severity, and inflammatory cell migration were significantly attenuated by melatonin in ethanol-fed mice. Moreover, serum and tissue inflammatory cytokines levels, tissue lipid peroxidation, and neutrophil infiltration were decreased and hepatocyte apoptosis was inhibited by melatonin treatment [84]. In addition, melatonin could inhibit ALT activity and oxidative stress. It was demonstrated that melatonin could also downregulate matrix metalloproteinases-9 and upregulate tissue inhibitor of metalloproteases (TIMP-1) expression in liver tissue. NFκB translocation into the nucleus induced by ethanol was significantly inhibited by melatonin [85]. Furthermore, Kupffer cells, cells isolated from ethanol-fed mice, would produce fewer ROS and TNF-α after melatonin treatment [84].

### 2.4. Protective Effects of Melatonin on Other Factor-Induced Liver Injuries

Radiation therapy is a popular and useful treatment for cancer [86]. However, ionizing radiation could interact with biological systems to produce excessive fluxes of free radicals that could impair a variety of cellular components [87]. Liver injury induced by radiation has been studied. After 12 h radiation exposure, both 8-OH-dG level and microsomal membrane rigidity were markedly elevated [88]. In addition, MDA and NO levels in the liver were significantly improved, and SOD and GSH-Px activity were reduced by whole body irradiation [86,89]. Melatonin scavenged free radicals directly, and exhibited benefits on liver injury induced by ionizing radiation [87]. The 8-OH-dG level and microsomal membrane rigidity were decreased, and hepatic MDA and NO levels were also decreased, while SOD and GSH-Px activities were considerably improved by pre-treatment with melatonin [86,88,89]. Melatonin fully counteracted the impairments produced by ionizing radiation. Except for ionizing radiation, liver injury could be caused by exposure to microwave radiation. Oxidative stress is the key mechanism of microwave-induced tissue injury [90]. Melatonin is a powerful antioxidant and could provide protection from liver injuries induced by microwave radiation. The increase in MDA induced by microwave radiation was decreased with melatonin treatment [90].

Liver failure subsequent ischemia-reperfusion (I/R) injury is recognized as a main difficulty in liver surgery [91]. Melatonin is a powerful endogenous antioxidant that possesses a protective role in liver I/R injury [92,93]. Melatonin protected the liver against I/R injury via overexpressing HO-1 [94]. Moreover, autophagy is related with production of ROS during I/R, and melatonin downregulated autophagy by activation of mammalian target of rapamycin (mTOR) signaling, which might in turn contribute to its protective effects in liver I/R injury [92].

Severe thermal injury may be complicated by dysfunction of organs distant from the original burn wound, including the liver, resulting in a serious clinical problem. The pathophysiology of burn-induced liver injury remains unclear, but increasing evidence suggests that the activation of inflammatory response, oxidative stress, endothelial dysfunction, and microcirculatory disorders could be the main mechanisms of hepatic injury [95]. Melatonin exhibited various biological activities, such as antioxidant and anti-inflammatory effects, and has been reported to display significant beneficial effects against burn-induced cellular injury [96]. In a burned-rat model, enhancement in hepatic MDA level (*p* < 0.001), vascular congestion, leukocyte infiltration around the central veins, intracellular vacuolization, hepatic cell degeneration, and apoptotic bodies were observed [97]. Moreover, elevated hepatic MDA was reduced (*p* < 0.01), and degenerative changes in the hepatocytes were restricted by administration of melatonin [97]. Moreover, hepatic NFκB expression, TNF-α level, plasma AST, and ALT activities were all enhanced by 2–3-fold at 24 h after burns [96]. Elevated hepatic NFκB activity and TNF-α were decreased significantly, and improved AST and ALT activities in plasma were suppressed (*p* < 0.001) by treatment with melatonin [96]. It could be concluded that melatonin protected against burn-induced liver injury by suppressing NFκB-mediated inflammatory response. In addition, thermal skin-induced injury triggered a marked enhancement in hepatic 4-hydroxynonenal (a main product of lipid peroxidation and mediator of oxidative injury). Melatonin ameliorated burn-induced liver injuries by increasing HO-1 expression, upregulating Nrf2 expression, decreasing the 4-HNE level, and reducing histopathological alterations in liver [98].

In addition to liver injuries induced by the abovementioned factors, melatonin had protective effects on other types of liver damage, which are summarized in Table 2.

**Figure 1:**
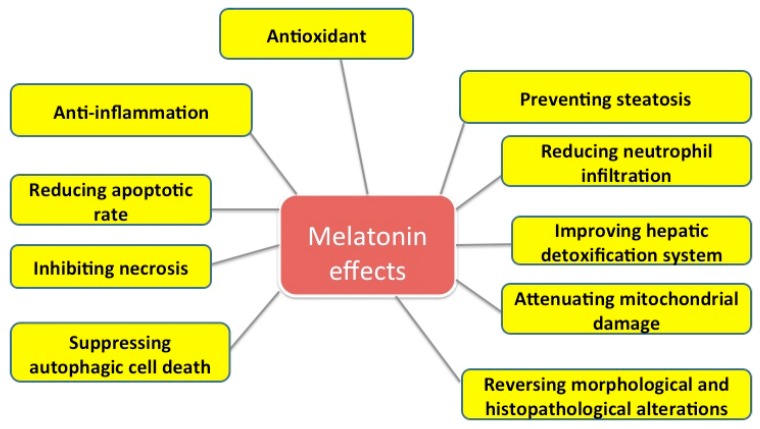
Some effects of melatonin on liver injuries.

Some effects of melatonin on liver injuries are summarized in Figure 1.

## 3. Protective Effects of Melatonin on Hepatic Steatosis

Liver steatosis is present in over two-thirds of the obese population. Hepatic steatosis could provoke insulin resistance and dysfunction of glucose and lipid metabolism [7]. Once steatosis has developed, the liver is “sensitized” to various inflammatory stimuli, which can precipitate nonalcoholic steatohepatitis [113]. However, there is a lack of effective treatment for hepatic steatosis. Recently, the role of melatonin in hepatic steatosis and its potential therapeutic effects have been identified.

A high-fat diet could induce oxidative stress with extensive liver steatosis in rats [7]. In rats fed a high-fat diet, mean liver weights (*p* < 0.001) and weight ratios of liver to body were reduced after melatonin treatment. Moreover, melatonin treatment significantly decreased hepatic steatosis. However, there was no evidence showing that melatonin reversed established steatosis [7]. Additionally, it has been demonstrated that melatonin has protective effects on hepatic steatosis induced by some other factors. Prenatal glucocorticoid overexposure could result in steatosis. In a prenatal glucocorticoid group, liver steatosis and apoptosis increased and the expression of leptin decreased. In addition, caspase 3, TNF-α, proteins expression, TUNEL stains, liver histone deacetylase, DNA methyltransferase activity, and DNA methylation were all increased in the prenatal glucocorticoid group. However, melatonin reversed these phenomena mentioned above and decreased liver steatosis [114]. In addition, estrogen deficiency and endoplasmic reticulum (ER) stress could also induce hepatic steatosis. In ovariectomized (OVX) rats, lipid accumulation and cellular oxidative stress were prevented by exogenous melatonin treatment in the liver. Melatonin alleviated steatosis and cellular oxidative stress in the livers of OVX rats [115]. Moreover, microRNAs (miRNAs) are pivotal regulators of gene regulation and their dysfunctions are common features in various metabolic diseases. Among miRNAs, miR-23a could regulate ER stress. Melatonin treatment rescued expression of miR-23a stimulated with tunicamycin, thus decreasing ER stress in primary hepatocytes and ameliorating ER stress-induced hepatic steatosis and inflammation [116].

## 4. Protective Effects of Melatonin on Non-Alcoholic Fatty Liver

Non-alcoholic fatty liver disease (NAFLD) may develop to end-stage liver diseases, which range from simple steatosis to steatohepatitis, advanced fibrosis, and cirrhosis. The main pathophysiological mechanisms of NAFLD are oxidative stress and lipid peroxidation [117]. Currently, there are no specific treatments against NAFLD [118]. NAFLD patients are characterized by hepatic steatosis, which several studies have demonstrated that melatonin attenuated [114,117]. Moreover, the effects of melatonin on NAFLD have been identified.

Some studies showed that melatonin protected against fatty liver mainly through preventing oxidative stress. Oxidative stress and extensive liver steatosis were observed in NAFLD rats, induced by a high-fat diet. Melatonin (2.5, 5, 10 mg/kg BW) improved SOD and GSH-Px activities, and a 10 mg/kg BW dose of melatonin decreased the MDA level in fatty liver. Additionally, melatonin (5 or 10 mg/kg BW) decreased hepatic steatosis and inflammation by lowering serum ALT, AST, liver total cholesterol, and TG in the fatty liver [117]. Another study determined the antioxidant activity of melatonin on hepatic oxidative stress in NAFLD female rats caused by ethionine. TG, MDA, and conjugate dienes (DC) were lower (*p* < 0.001), while GSH-Px activity was higher (*p* < 0.05) after treatment with melatonin. It could be concluded that hepatic oxidative stress in NAFLD female mice was reduced by melatonin [119]. In addition, melatonin reduced fatty liver by decreasing the level of pro-inflammatory cytokines and improving some parameters of fat metabolism in patients with NAFLD [120].

Diabetes mellitus patients were very likely to also have chronic liver disease. Moreover, chronic liver disease might be a leading cause of death in patients with diabetes mellitus. It was found that a majority of liver injuries induced by diabetes mellitus were associated with NAFLD [121]. Therefore, the protective effects of melatonin on diabetes mellitus-induced liver injury are also discussed in this section. Melatonin has been found to act as an anti-diabetic agent in animal models [122]. Melatonin improved glucose intolerance and insulin resistance in high fat diet-induced diabetic mice [123]. Moreover, melatonin was demonstrated to possess beneficial effects on liver injury induced by diabetes. The mechanism of protection might be associated with elevation in the antioxidant status of cells and mitochondrial physiology [124].

Diabetic rats were observed with markedly higher blood glucose levels than the rats of the control. Mean body weights of diabetic rats were meaningfully lower than those of the control. In histological investigations, hydropic and nuclear changes were observed in hepatocytes in the diabetic rats, and cellular glycogen depletion, congestion, sinusoidal dilatation, inflammation, and fibrosis were found in diabetic rats. In addition, both glycogen granules in the hepatocyte cytoplasm and mast cell granules were decreased in the diabetic rats [125,126]. Melatonin had a positive effect on these parameters. It was demonstrated that melatonin restored the morphological and histopathological changes of the liver induced by diabetes [127]. Additionally, MDA, protein carbonyl (PCO) and 8-hydroxy-2-deoxyguanosine (8-OHdG) levels in the plasma and the liver homogenates were considerably decreased due to melatonin administration. Total thiol (T-SH) and GSH levels in liver were meaningfully increased in diabetic rats following melatonin treatment [128,129,130].

Mitochondrial dysfunction and an overproduction in mitochondrial ROS during diabetes caused pathological consequences of hyperglycemia [124]. Moreover, the impairment of mitochondrial respiratory activity plays a key role in liver injury during diabetes [131]. The effects of melatonin on this particular functional impairment in rats’ liver mitochondria have been identified. In diabetic rats, the oxygen consumption rate V_3_ and the acceptor control ratio were reversed to those of non-diabetic rats by melatonin. In addition, the suppressed activity of CAT in the cytoplasm of liver cells was restored, and mitochondrial GST inhibition was prevented by melatonin [124]. Thus, melatonin might regulate mitochondrial function under diabetes.

## 5. Protective Effects of Melatonin on Hepatitis

Hepatitis is a critical clinical issue. The pathogenesis of hepatitis is various, including viruses, drugs, alcohol, toxins, and so on. Developing an effective therapeutic agent for hepatitis is urgent. There is evidence showing that melatonin possesses beneficial effects on hepatitis.

In several experimental models, some drugs, such as acetaminophen, amoxicillin-clavulanic acid, albendazole, and labetalol, could induce toxic hepatitis [132,133,134,135]. Some food supplements might also induce toxic hepatitis [136]. Interestingly, in intact animals, GSH concentration and activities of GSH-Px, GSSG-R, NADP-isocitrate dehydrogenase, and glucose-6-phosphate dehydrogenase increased after the administration of melatonin. However, in animals with toxic hepatitis, GSH concentration and these enzyme activities decreased after melatonin treatment, which was probably associated with an inhibition of free radical oxidation [137].

The effects of melatonin on fulminant hepatitis induced by rabbit hemorrhagic disease virus (RHDV) have been identified in rabbits. RHDV infection triggered an inflammatory response; meanwhile, toll-like receptor 4, high-mobility group box (HMGB)1, IL-1β, IL-6, TNF-α, and C-reactive protein expression were increased, while decay accelerating factor (DAF/CD55) expression decreased. Melatonin meaningfully restored those changes. Melatonin also lowered matrix metalloproteinase-9 expression. Moreover, RHDV infection inhibited the hepatic regenerative/proliferative response and decreased the expression of hepatocyte growth factor (HGF), epidermal growth factor, platelet-derived growth factor (PDGF)-B, vascular endothelial growth factor, and their receptors, which were inhibited by melatonin treatment. Additionally, melatonin reduced phosphorylated Janus kinase expression and enhanced extracellular mitogen-activated protein kinase (ERK) and signal transducer and activator of transcription (STAT) 3 expression. It has been shown that melatonin had an anti-inflammation effect and stimulated regenerative mechanisms in rabbits infected by RHDV [138]. Concomitantly, hepatocyte apoptosis was crucial in the progress of fulminant hepatitis infected by RHDV. Melatonin reduced apoptotic liver damage by attenuating ER stress via modulation of unfolded protein response signaling [139].

NAFLD might progress into nonalcoholic steatohepatitis, and the major process is oxidative stress with excessive production of ROS and inflammatory cytokine generation [140]. Patients with histological evidence (liver biopsy) of nonalcoholic steatohepatitis and no history of alcohol abuse were included to determine the effects of melatonin on nonalcoholic steatohepatitis. After three months’ treatment with melatonin, enzymes in the plasma and liver of the patients significantly improved without any side effects [140,141].

## 6. Protective Effects of Melatonin on Liver Fibrosis

Liver fibrosis is a wound-healing process of the liver in response to repeated and chronic liver injuries to hepatocytes or cholangiocytes. Based on the pathogenesis of liver fibrosis, therapeutic approaches to liver fibrosis could target each step of the process, including hepatocyte apoptosis, cholangiocyte proliferation, inflammation, and activation of myofibroblasts to deposit extracellular matrix [142]. Several studies have suggested that melatonin might be developed into a promising treatment for liver fibrosis. In addition, some studies demonstrated that melatonin attenuated liver fibrosis via limiting the expression of profibrogenic genes [143], directly suppressing hepatic stellate cells activation [144], and so on.

Hepatic fibrosis was commonly caused by CCl_4_ in experiments. In a study, it was demonstrated that melatonin attenuated CCl_4_-induced liver fibrosis through preventing necroptosis-associated inflammatory signaling. Melatonin reduced hepatic hydroxyproline content, hepatocellular damage, and transforming growth factor β1 and α-smooth muscle actin expression [145,146]. Moreover, melatonin significantly attenuated RIP1 expression, RIP1 and RIP3 necrosome complex formation, and mixed lineage kinase domain-like protein level in the liver [145]. Concomitantly, the expression of NFκB in the liver was inhibited, and the production of pro-inflammatory cytokines including TNF-α and IL-1β from Kupffer cells was decreased in fibrotic rats [147]. In another study, melatonin protected against liver fibrosis via inhibiting mitochondrial dysfunction, upregulating mitophagy, and mitochondrial biogenesis. Meanwhile, melatonin attenuated hallmarks of mitochondrial dysfunction, including mitochondrial swelling and glutamate dehydrogenase release [148]. In addition, pathologic evidence showed that melatonin prevented fibrosis (*p* < 0.05) caused by CCl_4_. AST, ALT, laminin, and hyaluronic acid levels in serum and hydroxyproline content in the liver were markedly lowered in the melatonin treatment group. Moreover, treatment with melatonin greatly decreased the MDA level and improved GSH-Px activity in the liver [149]. Additionally, a combination of melatonin and human dental pulp stem cells transplantation (hDPSCs) were better at suppressing liver fibrosis and restoring ALT, AST, and ammonia levels in the group of CCl_4_-injured mice than treatment with melatonin or hDPSCs alone [150].

Liver fibrosis could also be induced by bile-duct ligation, thioacetamide, and dimethylnitrosamine. Melatonin suppressed hepatic fibrotic changes (*p* < 0.001), lowered collagen, MDA, luminal, and lucigenin levels, and increased GSH levels in fibrotic liver caused by bile-duct ligation [151]. AST, ALT, and alkaline phosphatase (AP) had lower activity in fibrotic rats receiving thioacetamide followed by melatonin than rats receiving thioacetamide only. Moreover, melatonin lowered the levels of proinflammatory cytokines and oxidized glutathione, and increased the GSH level in the fibrotic liver. Additionally, an increase in the activity of paraoxonase 1 (PON-1) toward phenyl acetate and paraoxon was observed in the liver and serum after melatonin treatment [152]. In fibrotic rats induced by dimethylnitrosamine, fibrotic changes were suppressed by melatonin. Hydroxyproline and MDA levels were reduced, and GSH and SOD levels were elevated by melatonin treatment. Interestingly, there were no meaningful alterations in biochemical parameters when treated with melatonin only [153].

## 7. Protective Effects of Melatonin on Liver Cirrhosis

Liver cirrhosis is a critical stage of chronic liver diseases that can lead to liver failure, portal hypertension, and hepatocarcinoma [154]. In patients with liver cirrhosis, disturbances in serotonin and melatonin homeostasis were observed [155]. Moreover, primary biliary cirrhosis might be a pineal deficiency disease [156]. Thus, melatonin secreted by the pineal gland might exhibit protection on liver cirrhosis.

Constant oxidative stress could cause cell damage and fibrogenesis under liver cirrhosis [154]. Melatonin, as a powerful antioxidant, has been demonstrated to be beneficial in cases of liver cirrhosis. In thioacetamide-induced liver cirrhosis, oxidative stress with extensive tissue damage and increased α-smooth muscle actin expression were observed. Melatonin treatment showed protective effects on the oxidative stress-related changes, which suggested that melatonin prevented tissue damage and fibrosis in liver cirrhosis caused by thioacetamide [154]. In another study, secondary biliary cirrhosis was induced by bile duct ligation, and melatonin (20 mg/kg BW) was treated intraperitoneally for two weeks, starting 15 days after an operation. The data indicated that melatonin was useful for different tasks, including re-establishing normal liver enzyme concentration, decreasing the hepatosomatic and splenosomatic indices, restoring lipoperoxidation and the antioxidant enzyme level, and decreasing fibrosis and inflammation, thus weakening liver tissue injury in secondary biliary cirrhosis rats [157]. Concomitantly, melatonin concentration was meaningfully increased in the plasma by the oral administration of melatonin (10 mg), both under fasting and postprandial conditions, particularly in liver cirrhosis patients [158]. Herein, melatonin might be developed into a therapeutic agent for liver cirrhosis.

## 8. Protective Effects of Melatonin on Hepatocarcinoma

Cancer is a major public health problem and one of the leading causes of death [159,160]. Hepatocellular carcinoma (HCC), the main type of liver cancer (70%–80%), is one of the most common cancers and its incidence is growing worldwide [161,162]. In addition, HCC is one of the most lethal human cancers because of its high incidence and metastatic potential and the low efficacy of conventional therapies [163]. Surgery, radiotherapy, and chemotherapy are the major treatment modalities, but could induce certain side effects [164]. Epidemiological studies have suggested that antioxidant supplements might reduce the risk of cancer recurrence and cancer-related mortality [165]. Melatonin, a powerful antioxidant, showed protective effects on hepatocarcinoma. Its oncostatic effects on hepatocarcinoma were mainly due to its antioxidant, antiproliferative, and pro-apoptotic abilities.

Melatonin is an effective natural antioxidant that acts through different mechanisms to weaken the impairments of ROS [166]. In H4IIE hepatoma cells, the effect of melatonin on the hydrogen peroxide (H_2_O_2_)-induced activation of the mitogen-activated protein kinase (MAPK) and mTOR signaling pathways was identified. H_2_O_2_-induced activation of the extracellular signal-regulated protein kinases (ERK)1/2 and p38 MAPK, and some of their downstream targets, were strongly weakened by melatonin. H_2_O_2_-induced phosphorylation of Akt and the Akt substrate mTOR, a downstream target of mTOR action, and eIF4E-binding protein 1 (4E-BP1) were also weakened by melatonin. Upregulation of ERK1/2, p38, and Akt signaling by H_2_O_2_ were all accompanied by activation of Ras. Thus, melatonin acted to inhibit many of the H_2_O_2_-induced changes in the MAPK and mTOR signaling pathways, mainly via preventing Ras [166]. In addition, supplementation with isoquercitrin or melatonin reduced the oxidative stress-mediated hepatocellular tumor-promoting effect of oxfendazole. The number of glutathione *S*-transferase placental form (GST-P)-positive foci promoted by oxfendazole was prevented by the combined antioxidant isoquercitrin or melatonin treatment, and the area of GST-P-positive foci was suppressed by melatonin treatment. The mRNA expression of cytochrome P_450_, family 2, subfamily b, polypeptide 2 (Cyp2b2), and malic enzyme 1 were decreased in the isoquercitrin and melatonin treatment groups, and mRNA expression levels of Cyp1a1 and aldo-keto reductase family 7, member A3 were also decreased in the melatonin treatment group. Furthermore, the production of NADPH-dependent ROS was inhibited in vitro due to isoquercitrin or melatonin treatment. Co-administration of isoquercitrin or melatonin suppressed the hepatocellular tumor-promoting activity of oxfendazole in rats by decreasing ROS production and activating Cyps [167]. In addition, it has been demonstrated that melatonin had effects on circadian rhythms of LPO and antioxidants in N-nitrosodiethylamine (NDEA)-induced hepatocarcinogenesis. Alteration of circadian systems could cause cancer and affect its development; meanwhile, circadian rhythms were markedly altered in tumors and tumor-bearing hosts [168]. Circadian rhythm characteristics, such as acrophase, amplitude, and mesor of thiobarbituric acid reactive substances (TBARS), SOD, CAT, GSH-Px, and reduced glutathione were significantly changed in NDEA-treated rats [3]. The amplitude and mesor values of these antioxidant indices were significantly increased and the mesor values of TBARS were decreased after melatonin administration. Melatonin also reversed further delays in acrophase in NDEA-induced rats [169,170].

The proliferation of a variety of cancer cell lines was suppressed by melatonin, but only a few studies have focused on this ability of melatonin in hepatocarcinoma [171]. In a study, the effects of melatonin on the mouse hepatoma cell line HEPA 1–6, co-incubated with ethanol, and tamoxifen, respectively, were investigated. The antiproliferative activity of melatonin was exhibited from 640 μM to 3 mM dose-dependently, which was meaningfully higher (*p* < 0.01) than that with the solvent (ethanol) alone. The mechanism of antiproliferative effect of melatonin might be the prolonged activation of MAPK, which was activated by phosphorylation 15 min after induction with melatonin [172]. In HepG2 human HCC cells, melatonin possessed a dose- and time-dependent antiproliferative effect after its administration for two, four, or six days at 1000 or 2500 μM. The cell cycle altered with a rise in the number of cells in G_2_/M phase at both 1000 and 2500 μM melatonin concentrations, and S phase cell percentage had a significant increase at 2500 μM. Moreover, protein expression of MT_1_, MT_3_, and retinoic acid-related orphan receptor-α increased after melatonin treatment [161]. Additionally, the receptor antagonist luzindole was used to assess the melatonin effects on cell viability and proliferarion in HepG2 human HCC cells. A significant reduction in cell viability was observed after melatonin treatment (1000 and 2500 μM), and a meaningful decrease in cAMP level was detected at a dose of 2500 μM melatonin treatment, which was partly blocked by luzindole. Phosphorylated p38, ERK, and JNK expression was increased by both melatonin concentrations. ERK activation was completely abolished and cytosolic quinone reductase type-2 mRNA level was markedly improved in luzindole-treated cells. The data showed that the effects of melatonin on cell viability and proliferation in HepG2 human HCC cells were partly regulated via the MT1 membrane receptor, which also seemed to be associated with the melatonin modulation of cAMP and ERK activation [171]. Interestingly, the exposure to weak, extremely low frequency magnetic fields could also affect cancer progression. However, the cytoproliferative and dedifferentiating effects exerted by magnetic fields were prevented after 10 nM melatonin treatment in HepG2 cells [173].

Apoptosis resistance in HCC is an important factor in hepatocarcinogenesis and tumor progression, and causes resistance to conventional treatments [174]. Therefore, pro-apoptotic ability might be a key factor in treating HCC. Melatonin has shown its pro-apoptotic effect in many studies. Inhibitor of apoptosis proteins (IAPs) have exhibited an ability to resist apoptosis. Four members of IAPs (cIAP-1, cIAP-2, survivin, and XIAP) were overexpressed in human HCC tissue. Melatonin overcame apoptosis resistance by inhibiting survivin and XIAP via the COX-2/PI3K/Akt pathway in HCC cells. Inhibition of the growth of HepG2 and SMMC-7721 cells and promotion on apoptosis, accompanied by the downregulation of survivin and XIAP were found after melatonin treatment. Moreover, cIAP-1, survivin and XIAP, were related to the co-expression of COX-2 in human HCC specimens, and melatonin also decreased COX-2 expression and prevented Akt activation in HepG2 and SMMC-7721 cells [174]. In HepG2 HCC cells, melatonin treatment induced apoptosis with improved caspase-3 activity and poly (ADP-ribose) polymerase proteolysis. The pro-apoptotic effects of melatonin were associated with cytosolic cytochrome c release, upregulation of Bax, and induction of caspase-9 activity [175]. In another study, melatonin (10^−8^–10^−5^ M) showed a dose-dependent antiproliferative effect but no cytotoxic effect on hepatoma cell lines HepG2 and Bel-7402. Moreover, when combined with doxorubicin, melatonin meaningfully increased the effects of cell growth inhibition and cell apoptosis. The mechanism of cooperative apoptosis induction might be related to reduced Bcl-2 expression and improved Bax and caspase3 expression [176]. Previous studies have shown that melatonin elevated the effects of some chemotherapeutic drugs in HCC [177]. A study identified the roles of melatonin in ER stress-induced resistance to chemotherapeutic agents in HCC. Pre-treatment with tunicamycin (an ER stress inducer) significantly reduced the apoptosis rate produced by doxorubicin, while co-pretreatment with tunicamycin and melatonin drastically elevated the apoptosis caused by doxorubicin in HepG2 and SMMC-7721 cells. Additionally, phosphorylated Akt expression was decreased due to melatonin. Moreover, the C/EBP-homologous protein level was increased and survivin level was decreased by melatonin [177].

Except for the abovementioned effects, melatonin has other abilities that have been widely studied, such as autophagy, anti-invasion, antimetastasis, and anti-angiogenesis. In hepatoma H22 tumor-bearing mice, it was discovered that melatonin triggered an autophagic process by increasing Beclin 1 expression and inducing a conversion of microtubule-associated protein 1 light chain 3(LC3)-I to LC3-II, the protein related to the autophagosome membrane. Moreover, the phosphorylation of mTOR and Akt was inhibited by melatonin [178]. In addition, the autophagy induced by melatonin might be a potential strategy to potentiate melatonin’s apoptotic effects [179]. Extracellular matrix degradation by matrix metalloproteinases (MMPs) is related to cancer cell invasion, and it has been suggested that the inhibition of MMPs by synthetic and natural inhibitors might be of great importance in HCC therapies [180]. Melatonin exhibited anti-invasive and antimetastatic effects through preventing MMP-9 activity in various tumor types. More specifically, melatonin regulated the motility and invasiveness of HepG2 cells in vitro via a molecular mechanism that involved TIMP-1 upregulation and attenuation of MMP-9 expression and activity via NFκB signaling pathway inhibition [180]. In addition, melatonin showed anti-angiogenic features in the HCC cell lines. Angiogenic (*CCL2*, *CXCL6*, *IL-8*) and angiostatic (*CXCL10*) chemokine gene expression in two HCC cell lines was influenced by melatonin. Upregulation of *CCL2*, *IL-8*, and *CXCL10* genes in the HCC24/KMUH cell line, but downregulation of *CCL2*, *CXCL6*, and *IL-8* genes in the HCC38/KMUH cell line, and upregulation of *CXCL10* gene in both cell lines, were found after melatonin treatment at pharmacologic concentrations (1 and 100 μM) [181]. Furthermore, melatonin exhibited an anti-angiogenic activity in HepG2 cells through affecting the transcriptional activation of vascular endothelial growth factor, via hypoxia inducible factor 1 α (Hif1α) and STAT3 [182].

**Figure 2:**
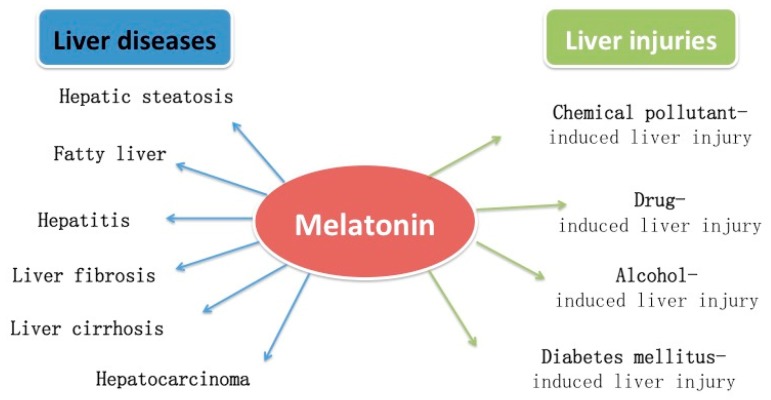
Protective effects of melatonin in several liver injuries and diseases.

**Figure 3:**
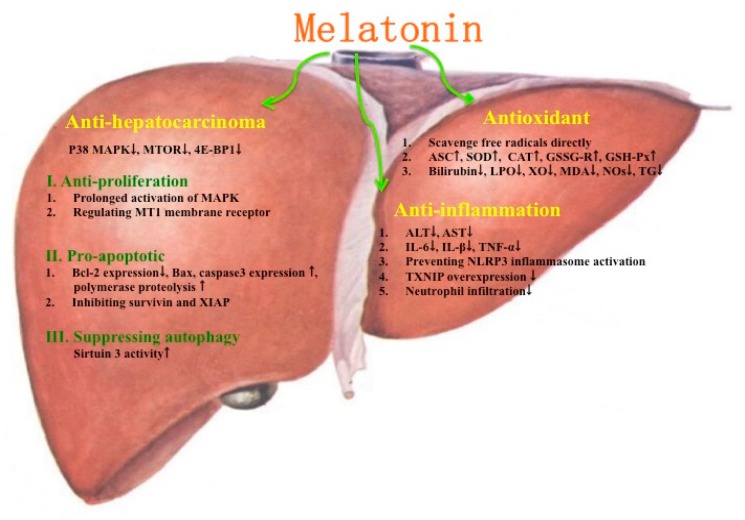
Some possible mechanisms of melatonin for improving liver injuries and diseases. ↑ stands for increase; ↓ stands for decrease.

The protective effects of melatonin on several liver injuries and diseases are summarized in Figure 2. Some possible mechanisms for melatonin improving liver injuries and diseases are given in Figure 3.

## 9. Conclusions

This review provides a detailed and updated description of the protective effects of melatonin against various factor-induced liver injuries and diseases. Melatonin has shown protective effects in liver injuries induced by chemical pollutants, drugs, and alcohol, as well as liver diseases including hepatic steatosis, fatty liver, hepatitis, fibrosis, cirrhosis, and hepatocarcinoma. Melatonin could alleviate liver injuries and diseases by preventing oxidative damage, improving mitochondrial physiology, inhibiting liver neutrophil infiltration, necrosis, and apoptosis, reducing the severity of morphological alterations, and suppressing liver fibrosis. However, related studies of melatonin applied to clinical treatment for liver injuries and diseases are limited. In the future, more clinical trials should be conducted to assess the effects of melatonin in this field. Furthermore, the mechanisms of action should be studied further.

## Figures and Tables

**Table 1:** The effects of melatonin on liver injuries induced by other toxins.

Toxins	Subjects	Methods of Melatonin Administration	Duration of Melatonin Treatment	Melatonin Doses	Melatonin Effects	Ref.
Methanol	Rats	Intragastric gavage	6 or 24 h	10 mg/kg BW or 3 g/kg BW	Reducing the MDA level significantly, restoring the protein carbonylation level, preventing the increase in nitrite level and MPO activity and the reduction in the antioxidant enzyme activities, and returning piecemeal necrosis, lobular lytic necrosis and portal inflammation to normal histologic appearances at a dose of 10 mg/kg	[46]
Fluoride	Mice	Peritoneal injection	30 days	10 mg/kg BW/daily	Preventing the decrease in body and liver weight as well as the decrease in liver enzyme activity of succinate dehydrogenase (SDH), acid phosphatase (ACP), alkaline phosphatase (ALP), and total liver protein level and diminishing the increase in serum glutamate oxaloacetate transaminase (SGOT) and serum glutamate pyruvate transaminase (SGPT) activities in the liver	[47]
Aluminum chloride	Rats	Oral administration	30 days	5 mg/kg BW/daily	Alleviating the increases in the plasma of the ALT, AST, ALP, total bilirubin, total lipids, total cholesterol, TG and glucose levels, and attenuating the decrease in total proteins, reducing oxidative stress, and improving histological changes	[48]
Dimethyl-nitrosamine	Rats	Intraperitoneal injection	14 days	50 mg/kg BW/daily	Improving serum and antioxidant enzyme activities, reducing the infiltration of inflammatory cells and necrosis in the liver, and increasing the expression of nicotinamide adenine dinucleotide phosphate (NADPH): quinone oxidoreductase-1, HO-1, and SOD2, and increasing novel transcription factor expression, nuclear erythroid 2-related factor 2(Nrf2) and decreasing inflammatory mediators expression	[49]
Thio-acetamide	Rats	Intraperitoneal injection	24 h	3 mg/kg BW	Decreasing serum liver enzymes and blood ammonia levels, improving liver histological changes, decreasing mortality of rats, inhibiting the increase in nuclear binding of nuclear factor kappa B (NFκB), and decreasing the hepatic level of thiobarbituric acid reactive substances, protein carbonyls and inducible NO synthase, improving survival and reducing liver damage and oxidative stress	[50]
Nicotine	Rats	Subcutaneous injection	30 days	10 mg/kg BW/daily	Attenuating the increase in LPO products and restoring the SOD activity and GSH level, and reducing both nitrotyrosine reactivity and tissue damage	[51]
Paraquat	Rats and hepatocytes	Preincubation with melatonin in vitro	30 min	0.5, 1 or 2 mM	Preventing in a dose- and time- dependent manner the loss of viability, the leakage of lactate dehydrogenase, depletion of intracellular glutathione and MDA accumulation, and inhibiting cell damage completely at 2 mM dose	[52]

**Table 2:** The effects of melatonin on other liver injuries.

Factors	Subjects	Methods of Melatonin Administration	Duration of Melatonin Treatment	Melatonin Doses	Melatonin Effects	Ref.
Liver resection	Patients	Through a nasogastric tube	A single dose	50 mg/kg BW	Resulting in lower postoperative transaminases, and inducing a trend toward shorter ICU stay and total hospital stay	[99]
Bile duct ligation	Rats	Injection or oral administration	8 days	500 μg/kg BW/daily, and 10, 100 mg/kg BW daily	Resulting a significant recovery of antioxidant enzymes and a reduction in the negative parameters of cholestasis at the concentration of 500 mg/kg, and attenuating cholestatic liver injury and reducing the increases in serum and hepatic TBARS concentrations and hepatic MPO activity at the concentration of 10 and 100 mg/kg	[100,101]
Hemorrhagic shock	Rats	Intravenous injection	A single dose	2 mg/kg BW	Normalizing liver Akt phosphorylation, increasing mTOR activation and HO-1 expression, and reducing cleaved caspase-3 level	[102]
Experimental hyperthyroid	Rats	Intraperitoneal injection	20 days	6 mg/kg BW/daily	Increasing the number of Kupffer cells, lipid vacuoles of Ito cells and microvilli of hepatocytes, and enlarging the spaces of disse	[103]
Hyperphenylalaninemia	Rats	Subcutaneous injection	From mating day until delivery	20 mg/kg BW/daily	Preventing the accumulation of LPO products	[104]
High cholesterol diet	Mice	Oral administration	4 months	10 mg/L in drinking water	Reducing plasma, liver cholesterol, hepatic MDA, diene conjugate (DC) and liver TG levels, increasing hepatic α-tocopherol and ascorbic acid levels and liver GSH-Px and GST activities, and attenuating the histopathological lesions	[105,106]
Constant light exposure	Rats	Subcutaneous injection	14 days	1 mg/kg BW/daily	Decreasing lipid peroxidation, and increasing GSH-Px activity	[107]
Intensive exercise	Rats	Intra-peritoneal injection	10 days	10 mg/kg BW/daily	Increasing the parameters of enzymes in serum, liver and kidney, and decreasing cellular degenerations	[108]
*Bacillus Calmette* Guerin and lipopolysaccharide	Mice, kupffer cells and hepatocytes	Using feeding needle in vivo or culture in vitro	10 days in vivo or 48 h in vitro	0.25, 1.0, 4.0 mg/kg BW/daily in vivo, 10^−9^, 10^−8^, 10^−7^, 10^−6^, 10^−5^ M in vitro	Decreasing serum ALT, AST activities at the concentration of 0.25, 1.0, 4.0 mg/kg, reducing MDA content, pro-inflammatory mediators (TNF-α, IL-1, NO) and immigration of inflammatory cells, upregulating SOD, attenuating the area and extent of necrosis and inhibiting TNF-α at the concentrations of 10^−8^–10^−6^ M, and decreasing IL-1 production of kupffer cells at the concentration of 10^−6^ M	[109]
*Opisthorchis viverrini*	Hamsters	Oral administration	30 days	5, 10, and 20 mg/kg BW/daily	Decreasing the formation of oxidative and nitrosative DNA lesions, 8-oxo-7, 8-dihydro-2’-deoxyguanosine, 3-nitrotyrosine and 8-nitroguanine in the nucleus of bile duct epithelium and inflammatory cells, reducing the HO-1 expression, mRNA expression of oxidant-generating genes (inducible NO synthase, NFκB, and cyclooxygenase-2) and proinflammatory cytokines (TNF-α and IL-1β), cytokeratin 19, nitrate/nitrite, 8-isoprostane and vitamin E levels, ALT activity and bile duct proliferation in the liver and increasing antioxidant genes (Nrf2 and Mn-SOD) expression	[110]
Rabbit hemorrhagic disease virus	Rabbits	Dissolved into dilutions	24 h	10 or 20 mg/kg BW	Inhibiting autophagic response significantly, and attenuating apoptosis	[111,112]
